# Structural and Optical Properties of Ag Nanoparticles Synthesized by Thermal Treatment Method

**DOI:** 10.3390/ma10040402

**Published:** 2017-04-12

**Authors:** Leila Gharibshahi, Elias Saion, Elham Gharibshahi, Abdul Halim Shaari, Khamirul Amin Matori

**Affiliations:** 1 Department of Physics, Faculty of Science, University of Putra Malaysia (UPM), Serdang 43400, Selangor, Malaysia; elhamgs2002@yahoo.com (E.G.); ahalim@upm.edu.my (A.H.S.); khamirul@upm.edu.my (K.A.M.); 2 Department of Physics and Astronomy, University of Texas at San Antonio, One UTSA Circle, San Antonio, TX 78249, USA

**Keywords:** Ag nanoparticles, conduction electrons, conduction bands, thermal treatment method

## Abstract

The modified thermal treatment method via alternate oxygen and nitrogen flow was successfully employed to synthesize very narrow and pure Ag nanoparticles. The structural and optical properties of the obtained metal nanoparticles at different calcination temperatures between 400 and 800 °C were studied using various techniques. The FTIR and EDX confirmed the formation of Ag nanoparticles without a trace of impurities. The XRD spectra revealed that the amorphous sample at 30 °C had transformed into the cubic crystalline nanostructures at the calcination temperature of 400 °C and higher. The TEM images showed the formation of spherical Ag nanoparticles in which the average particle size decreased with increasing calcination temperature from 7.88 nm at 400 °C to 3.29 nm at 800 °C. The optical properties were determined by UV-vis absorption spectrophotometer, which showed an increase in the conduction band of Ag nanoparticles with increasing calcination temperature from 2.75 eV at 400 °C to 3.04 eV at 800 °C. This was due to less attraction between conduction electrons and metal ions as the particle size decreases in corresponding to fewer numbers of atoms that made up the metal nanoparticles.

## 1. Introduction

Noble metal nanoparticles, in particular, Ag nanoparticles, are extensively studied because of their unique physical and chemical properties, which are different from those of the bulk metal [1,2,3,4,5]. Their properties are attributed to intra-band quantum excitations of the conduction electrons [6,7], mimicking the interactions of light on metal surface via the photoelectric absorption and Compton scattering. This makes Ag nanoparticles a very good candidate for technological applications, for instance in surface enhanced resonance Raman scattering [8,9], optical biosensor [10,11], and photo-catalysis [12,13]. Silver nanoparticles have biomedical and antimicrobial applications in human healthcare such as coating contact lenses, cardiovascular implants, wound dressing, disease diagnosis and treatment, bone cement and other implants, medical catheters, bandages, endodontic filling materials, dental instruments [14].

Different methods of synthesizing Ag nanoparticles by using inorganic salts as metal precursors have been reported, such as by chemical reduction [15,16,17,18,19], photochemical method [20,21,22,23,24], electrochemical method [25,26,27,28,29], microwave processing [30,31,32], ultra-sound processing [33,34,35], and gamma irradiation [36,37,38]. Most of these methods have achieved particles of the required size and shape, but they had some disadvantages, such as complicated preparation procedures, difficult to attain pure particles, and toxic by-products that may harm the environment. To overcome some of these shortcomings, the thermal treatment method is proposed here for the synthesis of metal nanoparticles for the first time. This method was intended to synthesize oxide nanomaterials, such as metal ferrite nanoparticles: ZnFe_2_O_4_, MnFe_2_O_4_, and CoFe_2_O_4_ [39,40,41]; metal chromic nanoparticles, ZnCr_2_O_4_ [42]; and semiconductor nanoparticles such as ZnO [43], CdO [44], TiO_2_ and ZrO_2_ [45,46]. By removing oxygen via nitrogen flow during calcination in an aqueous solution containing metal precursor and polyvinyl pyrrolidone (PVP), the metal nanoparticles can be manufactured using the thermal treatment method. Since no other chemicals were added into the reactant, the thermal treatment method has the advantages of simplicity, low cost, and is environmentally friendly as no toxic and unwanted products are discharged into the drainage system.

## 2. Results

### 2.1. Mechanism of Ag Nanoparticles Formation via Thermal Treatment Method

**Figure 1:**
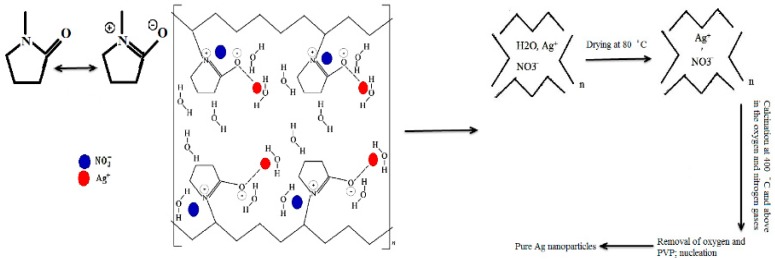
The mechanism of interactions between polyvinyl pyrrolidone (PVP) and silver ions in the formation of silver nanoparticles.

**Figure 2:**
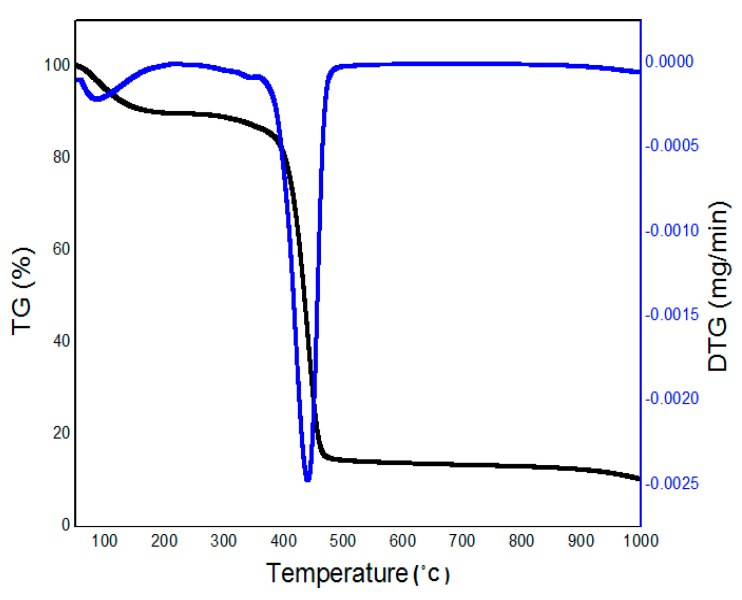
Thermogravimetric (TG) and thermogravimetric derivative (DTG) curves for PVP/Ag at a heating rate of 10 °C/min.

Figure 1 schematically shows the mechanism of Ag nanoparticles formation via thermal treatment method. The interaction between PVP as the capping agent and ions (Ag^+^) is strong by way of ionic bonds between the Ag ions and the amide group via oxygen in the PVP chain [47]. The PVP stabilized silver nitrate by means of amide group steric and electrostatic stabilization. The aqueous solution contains Ag^+^ and NO_3_^−^ ions and the amide group of PVP. Before the drying process, Ag^+^ ions could start to receive free electrons in the solution to form Ag^0^ metal atom (nucleation process). In the drying process at the temperature of about 80 °C (Figure 2), water started to evaporate and PVP started to decompose and shorten the polymer chains that cap Ag^0^ atoms and Ag^+^ ions [48]. In calcination with oxygen gas flow at temperatures from 400 to 800 °C, PVP decomposed into some gases such as CO, CO_2_, H_2_, and NO_2_, which can be removed or dissolved by passing the gases into a container filled with water. Ag nanoparticles start to grow after smaller Ag nanoparticles agglomerated into larger particles when PVP was removed. At the same time, Ag–O nanoparticles could form following the calcination under oxygen gas flow. Later, the calcination process continued under nitrogen gas flow to remove oxygen from possible Ag–O nanoparticles to produce the required pure Ag nanoparticles [49].

### 2.2. Thermal Analysis (TGA–DTG Measurement)

Thermogravimetric analysis and its derivative form (TGA–DTG curves) were used to study the degradation of the sample and to determine the appropriate starting temperature for the calcination process. Figure 2 shows the thermogram of percentage weight change as a function of temperature for the sample during calcination heating at 10 °C/min. It is evident that the sample started undergoing degradation process at the temperature of 85 °C because of the removal of moisture from the sample. At the temperature of 400 °C the PVP started to decomposed and at 436 °C, the main reduction of weight in the sample had occurred, in which the PVP almost decomposed completely, leaving Ag nanoparticles behind [43,50].

### 2.3. Phase Composition Analysis (FTIR Spectrosscopy)

A FTIR spectroscopy (Perkin Elmer, Waltham, MA, USA) helps analyze multi-component systems and provides necessary information pertaining the material’s phase composition and type of interactions existing between various compounds and polymers. In this study, FTIR measurement was employed to monitor the removal of PVP and to gauge the purity of Ag nanoparticles formed at various calcination temperatures. The FTIR spectra over the wave number range of 280–4000 cm^−1^, showed the organic and inorganic contents of the sample before calcination at 30 °C and after calcination at temperatures from 400 to 800 °C.

**Figure 3:**
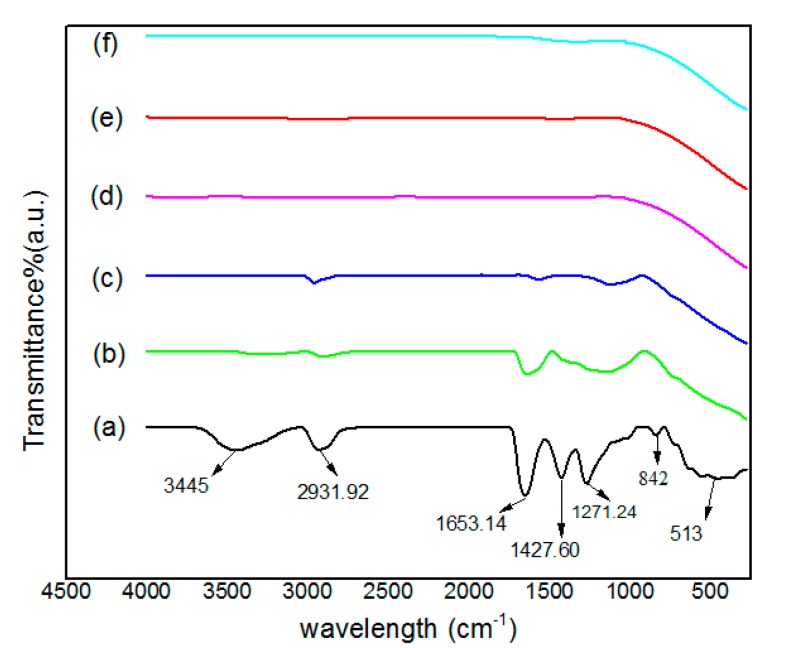
FTIR spectra of PVP and Ag nanoparticles at (**a**) 30; (**b**) 400; (**c**) 500; (**d**) 600; (**e**) 700 and (**f**) 800 °C in the range of 280–4500 cm^−1^.

Figure 3 shows all absorption peaks that belong to PVP and Ag nanoparticles. Before calcination, as shown in Figure 3a the absorption peaks at the wave number of 3445, 2931, and 1653 cm^−1^ belong to the vibration of the N–H, C–H and –C=C– stretching of covalent bonds, respectively. The absorption peaks with the wave numbers of 1427, 1271, and 842 cm^−1^ correspond to the C–C in the ring, C–N stretching and C–C in ring vibrations of covalent bonds, respectively. The peak at the 513 cm^−1^ indicates the vibration frequency of Ag–O ionic bond groups [51,52,53]. Figure 3b relates to the FTIR spectrum for the sample at the calcination temperature of 400 °C. Ag–Ag metallic bonds could already establish below 400 cm^−1^. The spectrum still shows the peaks after the wave number of 1000 cm^−1^. The peaks that locate at wave numbers of 3340, 2899, 1636, and 1125 cm^−1^ belong to the vibrations of the N–H, C–H, –C=C–, and C–N stretching bonds, respectively. These peaks belong to the organic and PVP bonds and it means incompletely removal of PVP from the sample in this calcination temperature. The peak that belongs to the vibration of the Ag–Ag metallic bonds cannot be seen in this graph because the FTIR uses the mid-infrared ray (4000–400 cm^−1^) that is not suitable to measure the vibration frequency of metal-metal bonds. Figure 3c–f show the FTIR spectrum of the samples for the calcination temperatures range between 500 and 800 °C. In Figure 3c, the spectrum includes very broad two peaks after 1000 at the wave numbers of 2962 and 1306 cm^−1^ that belong to the vibrations of C–H stretching and N–O symmetric stretching bonds that confirm the little presence of PVP and the organic part including oxygen in the sample. As shown in Figure 3d–f, by increasing the temperatures of calcination from 600 to 800 °C the spectrum does not show any peaks that confirm the complete removal of PVP and organic bonds including oxygen molecules from the samples. The vibrational spectra of Ag nanoparticles samples were not observed as absorption peak at any wave numbers during calcination at 500, 600, 700 and 800 °C. This evidence implies that by increasing calcination temperatures, higher purity of Ag nanoparticles have been obtained.

### 2.4. Elemental Composition Analysis (EDX Spectrosscopy)

**Figure 4:**
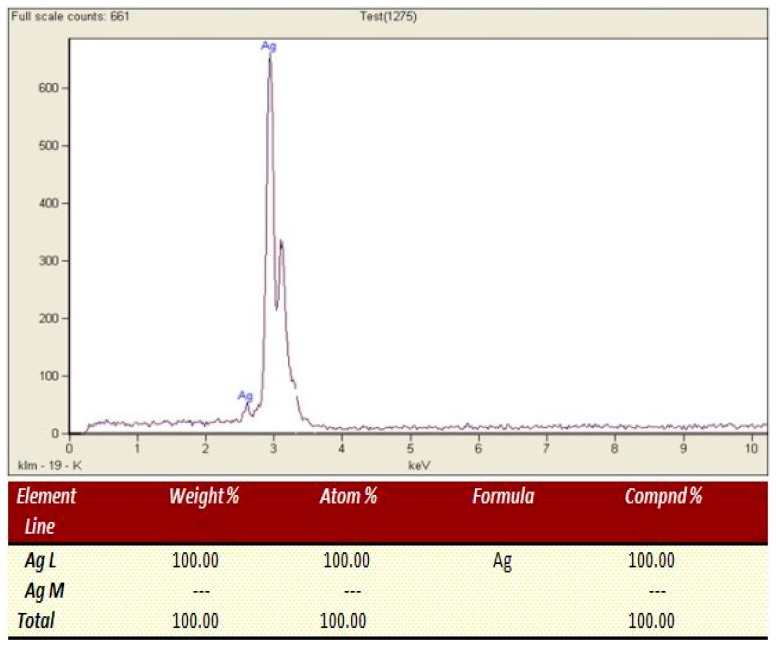
The EDX spectrum of the Ag nanoparticles calcined at 700 °C.

The elemental composition of the nanoparticles sample formed by thermal treatment method was determined by using energy dispersive X-ray (EDX) spectroscopy. EDX spectrum of Ag nanoparticles that calcined at 700 °C is illustrated in Figure 4. From the spectrum, the Ag element is present in the prepared sample as shown by its respective peaks. The recorded atomic percentage of Ag was 100.00%. This result indicated that the final product is pure Ag nanoparticles. The EDX analysis confirms that the pure silver nanoparticles were produced successfully by using modified thermal treatment method.

### 2.5. Structural Analysis (X-ray Diffraction (XRD))

**Figure 5:**
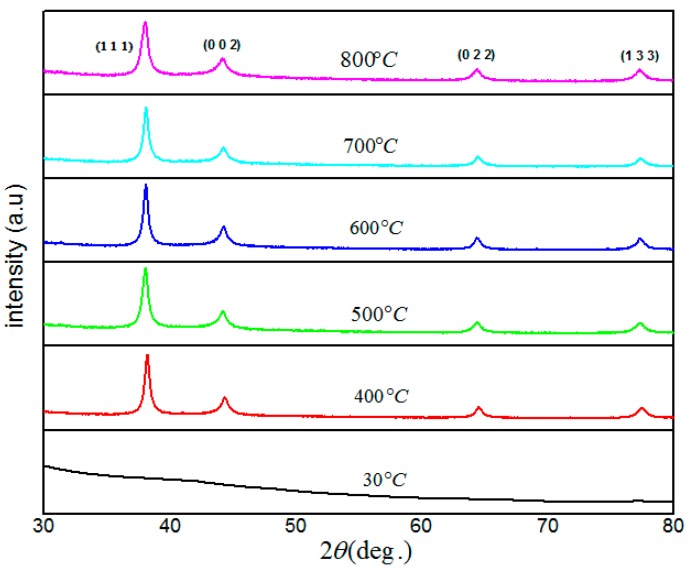
XRD patterns for Ag nanoparticles powder calcined at the temperature of 30, 400, 500, 600, 700, 800 °C.

Figure 5 shows the typical XRD patterns of the samples before and after calcination. The pattern of XRD for the sample before calcination at room temperature, which includes only PVP and silver nitrate and was drying at 80 °C for 24 h, shows no diffraction peaks. This means that the sample was amorphous (containing PVP, Ag^0^ and Ag^+^ ions) and no crystallized materials were found before calcination. For the calcined samples at 400 °C and above, the diffraction peaks imply that the formation of crystalline Ag nanoparticles have been established. The XRD patterns of calcined samples show the reflection planes of (1 1 1), (0 0 2), (0 2 2), and (1 3 3). The high-intensity peak in all samples was located at 2θ = 38.0°, which belongs to the reflection of crystal plane with Miller indices of (1 1 1). This high-intensity peak confirms the presence of Ag nanoparticles with a cubic structure in all samples at different calcination temperatures. The result is well matched with the standard phase reported in the XRD database with reference code 98-006-2693 of silver crystals in cubic structure with *a* = 4.0880 Å and volume = 68.32 Å.

From XRD spectra, it is apparent that the diffraction peaks become broad by increasing the calcination temperature, since the size of particles decreased by increasing the calcination temperature (Table 1). The samples’ crystallite sizes were found to range from 5.16 to 3.65 nm, which were calculated by using Scherer equation from the full width at the half maximum (FWHM) peak broadening of the high-intensity peak, (1 1 1) of the XRD graphs:

(1) $$D\left(nm\right)=\frac{k\lambda }{\beta \cos\theta }$$

where *D* is the crystalline size (nm); *k* is the shape factor, which is equal to 0.94 for sphere particles; β is the full width of the diffraction line at half of the maximum intensity measured in radians; λ is the X-ray wavelength of Cu Kα = 0.154 nm; and θ is the Bragg angle.

### 2.6. Morphology and Size Distribution (TEM Images for Ag Nanoparticles)

**Figure 6:**
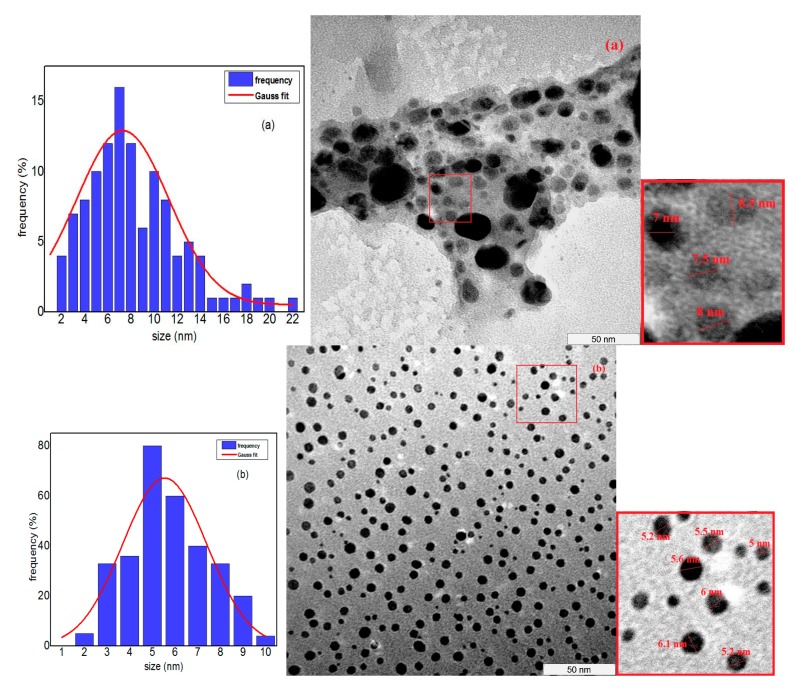

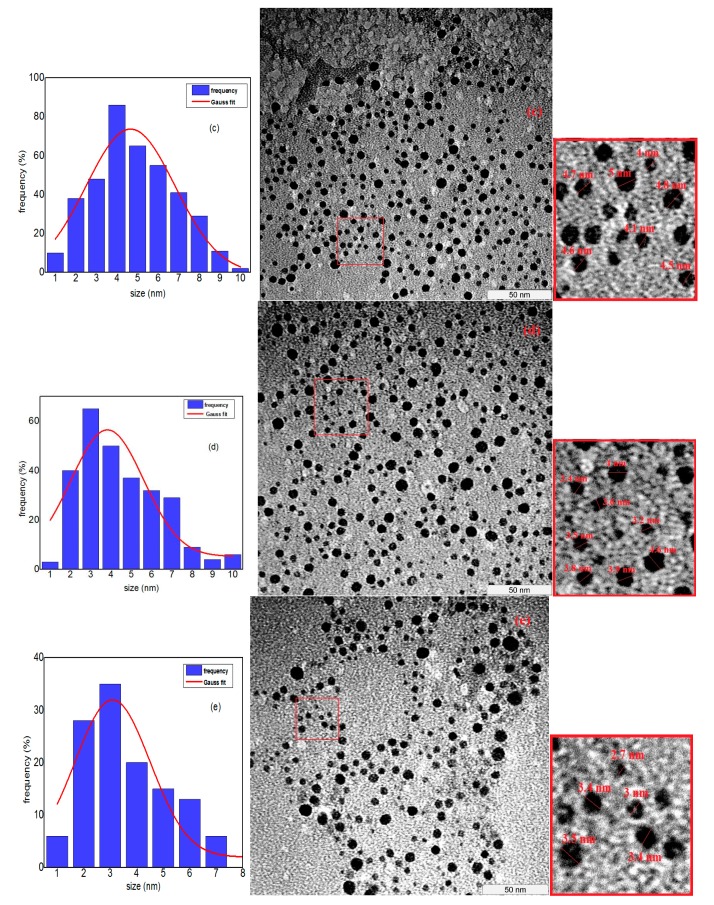
TEM image and particle size distribution of samples calcined at (**a**) 400; (**b**) 500; (**c**) 600; (**d**) 700 and (**e**) 800 °C.

The aims of using TEM spectroscopy are to investigate the morphological structure and average size distribution of the Ag nanoparticles. TEM images, as shown in Figure 6a–e, illustrate that Ag nanoparticles are almost spherical in shape and have uniformity in the morphology and size distribution for all samples that calcined in different temperatures. The average size and the standard division of the nanoparticles were achieved by using the Image Tool software, which gives the diameters of the particles in nanometer according to the scale bar indicated in the enlarged images. Furthermore, the graph of the size distribution of the nanoparticles in each image was drawn using Origin 9 software and fitting with the Gaussian function, which clearly indicated the size decreasing due to the increase in temperature. The unified scale of all images is 50 nm, which is shown in the bottom right of the TEM images.

The average particle sizes at calcination temperatures of 400, 500, 600, 700 and 800 °C are about 7.78, 5.57, 4.61, 3.75, and 3.29 nm, respectively, which were found to be in good agreement with the XRD measurements. These results show that by increasing the calcination temperature, the average particles sizes are decreasing.

The nucleation of Ag nanoparticles already formed even before the calcination at 400 °C. During the thermal treatment, heat caused the release of electrons from medium materials and more Ag atoms were formed. Heat also made the PVP decompose and slowly released the capping property on Ag atoms and Ag particles. Thus Ag particles tend to agglomerate to form larger Ag nanoparticles. By increasing the calcination temperatures, due to the Oswald ripening process, the less bonded PVP on the particles’ surface decomposed and the particles agglomerated to enlarge the particle size [54,55,56]. On the other hand, since Ag nanoparticles are surrounded by conduction electrons, the repulsive force prevents Ag nanoparticles from agglomeration further. As the temperature of calcination increased, the thermal vibration and mobility of the particles increased as well, which prevents further agglomeration of the particles. Due to increasing thermal vibration and repulsive action of conduction electrons surrounding Ag nanoparticles, the particle size was limited to less than 8 nm when compare to more than 10 nm for semiconductor nanoparticles such as ZnO [43], CdO [44], TiO_2_ [45] and ZrO_2_ [46] synthesized by thermal treatment method. Moreover, the particle size of the quantum dots increased with increasing calcination temperature due to particle agglomeration. Conversely, by increasing the calcination temperature, the particle size of Ag nanoparticles effectively decreased. The thermal vibration and repulsive action of Ag nanoparticles are the most likely reasons of decreasing particle size with increasing calcination temperature, as the evaporation of Ag particles cannot take place at the applied temperatures, since the melting and boiling temperatures of silver are 960.8 and 2212 °C respectively [57]. Furthermore, according to the TGA result, the main weight loss of the sample had occurred at 436 °C, which related to PVP decomposition, and at higher temperatures, the weight was not changed, indicating that the evaporation of silver did not happen up to 1000 °C.

### 2.7. Optical Properties

**Figure 7:**
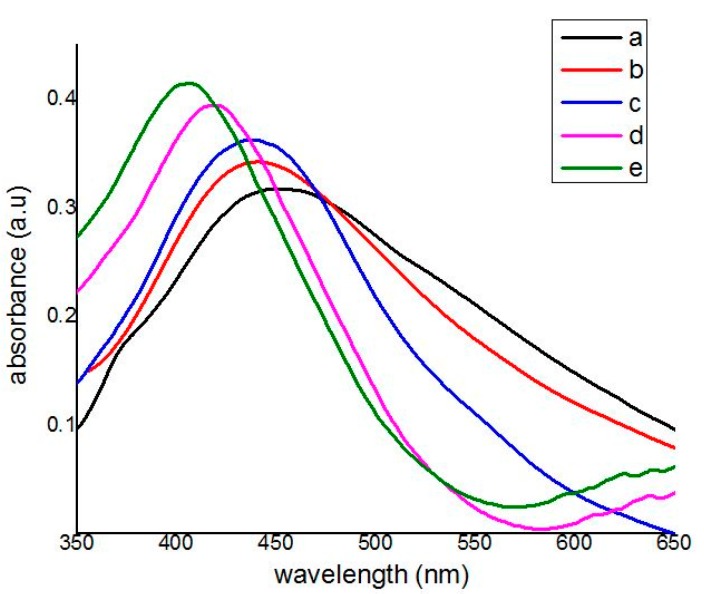
UV-visible absorption spectrum of Ag nanoparticles synthesized at the different calcination temperatures, (**a**) 400; (**b**) 500; (**c**) 600; (**d**) 700; (**e**) 800 °C.

The optical absorption spectra are normally determined by a UV-visible spectrophotometer. The function of UV-visible absorption is based on measuring the intensities of two transmitted beams, one beam transmitted from the sample and another transmitted from the reference cavity. In order to investigate the calcination effect on the optical properties of Ag nanoparticles, all calcined samples were taken from the dispersion of Ag nanoparticles in two percent PVP solution and measured the absorption spectra in the range of 200–800 nm at room temperature, which are as shown in Figure 7.

It is clear from the absorption spectra that the maximum absorbance wavelengths (λ_max_) blue shifted from 450 to 407 nm by increasing the calcination temperatures from 400 to 800 °C. This blue shifting is due to the decreasing in size of the nanoparticles by increasing the calcination temperatures. The maximum absorbance wavelength is associated with the conduction band energy according to quantum theory of metal nanoparticles [6,58,59].

**Figure 8:**
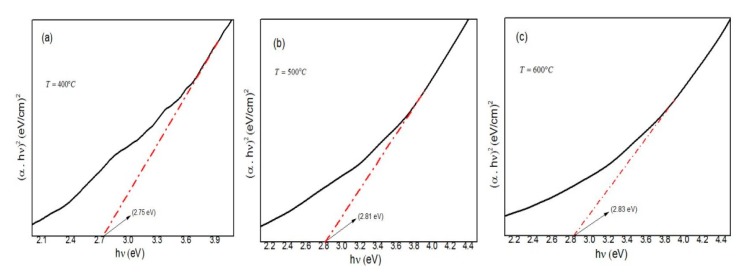

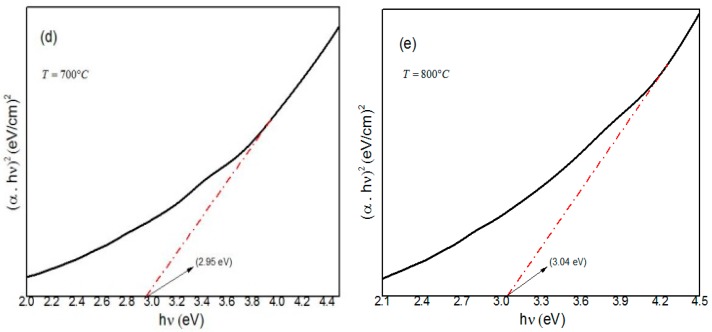
Tauc plot of Ag nanoparticles calcined in different temperatures. (**a**) 400; (**b**) 500; (**c**) 600; (**d**) 700; and (**e**) 800 °C.

The conduction band energy, *E*_cb_ (eV) can be calculated directly from the UV-visible absorption spectra by using the following Einstein’s photon energy equation:

(2) $$E_{\mathrm{cb}\;}=\frac{hc}{\lambda_{\max}}$$

where λ_max_ is the maximum absorbance wavelength, *h* is the Planck constant and *c* is the speed of light. The results are listed in Table 2. Since it can be seen that some of the absorption spectra have a wide peak, therefore, the conduction band energy of Ag nanoparticles can also be calculated indirectly from the absorption spectra by the following Tauc’s equation:

(3) $${\left(\alpha h\nu \right)}^{2}=B\left(h\nu -E_{\mathrm{cb}\;}\right)$$

where α is the absorption coefficient, *hv* is the photon energy, *E*_cb_ is the conduction band energy, and *B* is a constant. According to this equation, by plotting the (α*hν*)^2^ versus (*hv*) and extrapolation of the linear part of the curve to the energy axis, the conduction band energy of Ag nanoparticles can be achieved as shown in Figure 8 [59,60]. It is obvious from the Figure 8 that by increasing the calcination temperatures from 400 to 800 °C the conduction band energy increased from 2.75 to 3.04 eV, respectively. These results as listed in Table 2 are in a good agreement with the direct calculations from Einstein’s photon energy Equation (2). 

The optical absorption of metal nanoparticles has been described traditionally and classically by Mie theory [61] as the localized surface plasmon resonance (LSPR). There is evidence that the optical absorption of metal nanoparticles can be described quantum mechanically due to intra-band excitations of conduction electrons by photon [6,58], mimicking the interactions of light on metal surface via the photoelectric absorption and Compton scattering. In metal nanoparticles, the conduction electrons are not entirely free as in the bulk structure, but instead some are held by the individual atoms and some are free and moved between atoms to form metallic bonds that cement the metal nanoparticles. Upon receiving photon energy of UV light having the maximum absorbance wavelengths (λ_max_), the conduction electrons experience intra-band quantum excitations beyond the Fermi energy level, from which the conduction band of metal nanoparticle is defined. For smaller particle size, fewer numbers of atoms form the particle and so reduce the potential attraction between the conduction electrons and metal ions of the particle. In this way, the conduction band energy increases for the smaller particle. Conversely, for larger particle size, large numbers of atoms form the particle, thus increasing the potential attraction between conduction electrons and metal ions and therefore reduce the conduction band energy of the metal nanoparticles [6].

## 3. Materials and Methods

Silver nitrate reagent (*M* = 169.88 g/mol) was used as the metal precursor, polyvinyl pyrrolidone (PVP) (*MW* = 58,000) was used as the capping agent, and deionized water was used as the solvent. All chemicals were purchased from Sigma-Aldrich (St. Louis, MO, USA) as analytical grade products and were used without further purification. The PVP solution was prepared by dissolving 2 g of PVP in 100 mL of deionized water at room temperature before 50 mg of silver nitrate was added and was stirred for 3 h. At the end of stirring, a colorless and transparent solution was obtained. No precipitation was observed in the solution. The solution was poured into a glass Petri dish and heated in an oven at 80 °C for 24 h to evaporate the water and form a solid cake, which was then crushed and ground in a mortar to form a uniform powder. The calcination of the powder was conducted at the temperature between 400 and 800 °C by 100 °C step, firstly in oxygen flow for 3 h to decompose the organic compounds, and secondly in nitrogen gas flow for 3 h to remove oxygen from oxides before producing pure Ag nanoparticles. Both gases were allowed to flow in succession into the calcination chamber at the same flow rate of 50 mL/min.

Several characterization techniques were used to investigate the synthesized Ag nanoparticles. The crystal structure of Ag nanoparticles has been examined by X-ray diffraction (XRD Shimadzu model 6000, Shimadzu, Tokyo, Japan) using Cu Kα (0.154 nm) as an X-ray source to generate diffraction patterns from the crystalline samples at ambient temperature in the 2θ range of 10°–80°. The infrared spectra (280–4000 cm^−1^) have been recorded using Fourier transform infrared spectrometer (FTIR Perkin Elmer model 1650, Perkin Elmer, Waltham, MA, USA) to monitor the removal the capping agent after calcination. The energy dispersive X-ray analysis (EDX, model Jeol Jsm 7600, Jeol, Tokyo, Japan) was used to analyze and identifying the elemental compositions of the sample after calcination at 600 °C. Both EDX and FTIR results were used to demonstrate the formation of pure crystalline Ag nanoparticles at various calcination temperatures. The morphology, particle size, and particle size distribution of the nanoparticles was determined using transmission electron microscopy (TEM model Hitachi H-7100 TEM, Hitachi, Tokyo, Japan) with an accelerating voltage of 500 kV. The sample was prepared on the copper grids by drying a drop of Ag nanoparticles powder dispersion in deionized water. Moreover, UV-vis spectrophotometer (Shimadzu model UV-3600, Shimadzu, Tokyo, Japan) was used to evaluate the optical properties of the calcined samples at room temperature in the range of 200–800 nm.

## 4. Conclusions

Ag nanoparticles have been successfully synthesized via simple thermal treatment method by using only silver nitrate, PVP, and deionized water as the ingredients. The calcination process in the oxygen and nitrogen gas flow enables the removal of organic compounds and produces the pure crystalline Ag nanoparticles. The absorbance wavelength of Ag nanoparticles blue-shifted from 450 to 407 nm, corresponding to the decreasing average particle size from 7.88 to 3.29 nm by increasing the calcination temperature from 400 to 800 °C. The conduction band energy increased from 2.75 eV at 400 °C to 3.04 eV at 800 °C, due to there being less attraction between conduction electrons and metal ions for the smaller particle size. The thermal treatment method has been successfully used to produce pure Ag nanoparticles, which has the advantages of simplicity and being environmentally friendly, as it produced no toxic and unwanted products into the drainage system.

## Figures and Tables

**Table 1:** Structural properties of synthesized Ag nanoparticles at different calcination temperatures.

Temperature (°C )	2θ (Deg.) ± 0.01	FWHM (Deg.) ± 0.01	*D*_XRD_ (nm)	*D*_TEM_ (nm)
400	38.09	1.70	5.16	7.88 ± 4.50
500	38.08	1.87	4.70	5.57 ± 1.75
600	38.08	2.04	4.30	4.61 ± 1.96
700	38.05	2.38	3.69	3.75 ± 2.23
800	38.04	2.40	3.65	3.29 ± 1.84

**Table 2:** Optical properties of synthesized Ag nanoparticles at different calcination temperatures.

Temperature (°C)	*D*_TEM_ (nm)	Absorbance Wavelength (nm)	Conduction Band (eV) by Equation (2)	Conduction Band (eV) by Equation (3)
400	7.88 ± 4.50	450	2.75	2.75
500	5.57 ± 1.75	441	2.81	2.81
600	4.61 ± 1.96	438	2.83	2.83
700	3.75 ± 2.23	420	2.95	2.95
800	3.29 ± 1.84	407	3.04	3.04
